# The Teeth a Test of Age, Considered with Reference to the Factory Children

**Published:** 1847-06

**Authors:** Edwin Saunders

**Affiliations:** The Baltimore College of Dental Surgery; M. R. C. S., England, and Surgeon Dentist to St. Thomas' Hospital; Fellow of the Medico-Chirurgical Society; Fellow of the Medico-Botanical Society; of the London Medical, and Westminster Medical Societies, &c. Corresponding Member of the New York Historical Society; Author of "Advice on the Care of the Teeth," &c.


### ARTICLE III.

*The Teeth a Test of Age, considered with reference to the Factory Children; addressed to the Members of both Houses of Parliament.* By EDWIN SAUNDERS, D. D. S., of the Baltimore College of Dental Surgery; M. R. C. S., England, and Surgeon Dentist to St. Thomas' Hospital; Fellow of the Medico-Chirurgical Society; Fellow of the Medico-Botanical Society; of the London Medical, and Westminster Medical Societies, &c. Corresponding Member of the New York Historical Society; Author of "Advice on the Care of the Teeth," &c.

THE renewed appeal which is now about to be made on behalf of the children employed in factory labor, affords another opportunity for the discussion of any subject connected with that interesting question, and for the investigation of any point which shall tend to facilitate the operation of whatever enactments shall be determined upon by the legislature. Such a subject appeared to present itself in the discovery of those physiological phenomena which may be regarded as the most certain signs by which, in the absence or independently of registration, the age of a child may be determined; possessing, as it does, a most importanti nfluence in any legislative enactments of this nature, in securing to the objects for whose protection they are designed, the full benefit of their provisions. The interesting inquiry on which the following observations are founded, originated in an application to several members of that department of the medical profession to whom the care of those organs more especially belongs, in the number of whom the writer had the honor to be included, requesting an opinion upon the practicability of ascertaining the age of children, from seven to fourteen years, inclusive, by the teeth.

The suggestion is due to a passage in one of Mr. Horner's letters,* in which, after considering the various plans which have been from time to time proposed for removing the practical difficulties in the application of the law, arising from the ab-

* See "Evils of the Factory System," by C. Wing, Esq.

sence of any certain criterion of age, he observes: "Some surgeons have laid great stress upon the development of the teeth, as a safe guide; and if the object were the ascertaining of the *actual age* of the child, such a test would, perhaps, be less liable to error than that of height, but as an evidence of bodily strength it is obviously not to be depended upon."

The statement contained in the latter part of this sentence will naturally excite surprise, being totally at variance with the testimony of the eminent members of the medical profession, as given in evidence, as well as with the experience and judgment of physiologists generally. And not the least surprising circumstance connected with the avowal of such a principle is, that it is in direct opposition to the spirit and intention of the act framed for the amelioration of the condition of the factory children, who are thus, to a great extent, deprived of the benefit it was designed to impart. Agreeably to the testimony of individuals the most competent, from the great extent of their knowledge and experience in such matters, it is highly desirable that a child should not be subjected to severe labor until a certain progress shall have been made in the development and maturity of its frame, and this without respect to absolute size and weight, which can only occur within a certain period from its birth; and upon this principle it is enacted, that below the age thus determined as that at which labor may be borne without injury to the system, children shall not be so employed. It is quite obvious, indeed, that so long as the formative processes are in full and active operation, anything which shall tend to produce excessive waste in the system must be injurious, both from the increased nutrition required for its reparation, and from the impaired condition of the digestive organs which it induces, not only by diverting the stream into other channels, but also, by diminishing the supply at the fountain.

Upon the principle, however, on which Mr. Horner's calculations proceed, all the evidence on this subject goes for nothing, and he does not hesitate to declare it as his opinion, that the actual age of the child is not the principal consideration in the regulation of factory labor. The qualification of physical size and general appearance of robustness and health, is certainly a very specious one; but as a test of strength, and capability of intense and continued labor, its operation must be prejudicial, and even cruel in the extreme. It is the opinion of the most distinguished physiologists, that a robust and apparently strong child, of large general developments, so far from being more capable of severe and sustained exertion than a more delicate one, is less so, on account of the increased nutrition necessary to carry on the growth, and that such a child would probably sustain more constitutional injury than the other, *ceteris paribus*, from the same amount of labor.

It is an universal law of nature, obeyed in all her kingdoms, to be traced through her entire economy, that her operations are all carried on in time, and that certain periods must elapse, varying in each class and species, but constant in the individuals of each, for the maturity of her creations, before they are capable of displaying their qualities, or of entering upon their functions. The individuals of each class may differ from each other in actual size, and premature appearances may be sometimes presented; the processes of formation and growth, however, are continued for an uniform period, and are found, on closer investigation, to admit of less variation than these external indications seem to imply. If this view be correct, the conclusion to which Mr. Horner arrives is equally fallacious; and, on his own admission, the teeth being a much better criterion of age than the height, and the height having been hitherto considered the best that could be employed, it follows, of necessity, that the development of the teeth must be regarded as the most valuable test that has yet been suggested.

But the evidence of the eminent physicians and surgeons who were examined upon this point is still in the recollection of the reader, and it is unnecessary to dilate upon the fallacy of such a view, more especially as it will again come under consideration in the course of this inquiry. It is quite obvious that to proceed on such principles is to neutralize, to a great extent, the operation of any enactments upon the subject, and to render them fruitful sources of the most injurious errors.

Perceiving at once the value of the suggestion, the author of the "Evils of the Factory System" immediately sought opinions from those whom he considered, from their pursuits and general acquaintance with the subject, to be the best qualified to offer an opinion upon its practicability.

How far are the teeth to be relied on as a criterion by which the age of children, from seven to fourteen years inclusive, may be ascertained? was the interesting inquiry which was now presented to the consideration of some members of that department of the profession more especially devoted to that branch of study. And although possessed of the deepest interest as a subject of physiological research, and therefore well deserving the most minute and patient investigation, yet it is one confessedly neglected, and to which no definite or decisive answer has been returned. The period has now, however, arrived, when it especially claims the attention of every humane and inquiring mind, and demands the most immediate and rigorous scrutiny. Associated, as it now stands, with the well-being, with the health and life, of a very considerable portion of the British youth of both sexes, it addresses itself not less to the heart of the philanthropist than to the inquiring mind of the physiologist. In this state of the question, it is presumed that any attempt to elucidate the subject, and to contribute materials from which something like an accurate conclusion may be ultimately arrived at, will meet with indulgence, as tending at least to excite inquiry, and thus leading to a more accurate and intimate acquaintance with its general character and features.

To be fully convinced that the establishment of some more accurate test than that which has been hitherto employed, is absolutely necessary to the efficiency of any legislative enactments for the protection of the little sufferers employed in this species of industry, it is only necessary to take a brief review of the measures which have been at various times adopted, and of the causes of their almost total failure. Such a history will exhibit, in a sufficiently clear and striking light, the value and importance which such a criterion would possess, when once fully established.

The extreme cruelty and physical detriment arising from the employment of young children in the cotton and other factories, submitted, as they were, to the labor which adults had formerly undergone with difficulty, respiring an atmosphere rendered impure, and even noxious, by imperfect ventilation, by the numbers congregated in a small space, and by the numerous artificial lights which, for a longer or shorter period of each day's labor, are abstracting the vital principle of the air, in addition to the enervating effects produced upon the frame by being constantly confined in a warm temperature—had long since excited the commiseration of the humane and benevolent, and had induced them to call for the interference of the legislature. Various causes have, however, contributed to prevent what has been hitherto done from being either efficient or satisfactory. Some of these have arisen from inherent imperfections in the acts themselves, their too restricted application, and last, though not least, from the difficulties which the present system of registration opposes in determining the age of the children, and which, from the total failure of the standard now in use—viz. the height, it is the object of the present inquiry to endeavor to obviate.

The splendid improvements which were introduced into the machinery employed in this species of manufacture, in the early part of the eighteenth century, in this country, succeeded each other with such rapidity as to threaten an immediate and total change in that department of our commerce. The magnificent results of the ingenious inventions of Arkwright, Hargreaves, and others, whilst they added immensely to the commercial advantages of the country, at the same time effected by no means an agreeable change in the condition of the operatives. The masters were thus enabled to conduct their establishments and their operations on a scale altogether unprecedented, and still unequalled in extent; to keep pace, however, with the increased velocity of the processes now accomplished by the machine, it was necessary that the workman should conduct his operations at an increased rate of speed, and that his hours of labor should be prolonged. Thus, by those very improvements which reflect so much credit on the genius and industry of the country, the artizan, who had hitherto conducted his operations in his own cottage, and carried his production to the market, for which, from the demand being generally somewhat in advance of the supply, he regularly received a remunerating price, was now compelled to forsake his little home, with all its comforts and joys, where his working hours were self-imposed and regulated by his capability, and to labor with an intensity and for a period far beyond that which he had hitherto experienced, to an extent, indeed, to which at first he found himself physically inadequate. Nor was he allured to this increased amount of labor, and surrender of comfort and comparative independence, by the prospect of a proportional increase of remuneration. The proprietors, having at considerable cost erected their machinery, and being led to expect that, at no distant period, they would be enabled to dispense almost entirely with adult labor, considered themselves in a position to make their own terms with the workmen, and to impose the labor, and to fix the remuneration, according to their own disposition or ability. It was not surprising that, in such a state of things, dissatisfaction should be felt in the minds of the workmen, and that a disaffection on the part of the latter towards their employers should arise. Thus, the men became refractory; combinations were formed, and demands were made, to which the masters felt no inclination to accede. Hostile feelings being once excited between the employer and the employed, and the former being rendered daily more and more independent of adult labor, the experiment was at once resolved upon, and instantly made, of employing children in the factories. The success of the experiment was complete; and supplies of children were thus furnished from the workhouses of the metropolis, Edinburgh, and various parts of the kingdom; the consequences were, of course, terrific to the operatives, who were not less surprised at the success of the experiment than they were dismayed at their own prospects. The proprietors were now enabled to impose their own terms, and to make their own arrangements; and the children, being renumerated according to the quantity of labor performed, or rather of the product of their labor, animated with all the buoyancy of youth, and incited by the prospect of increased gain, exerted themselves to the very utmost that their frames were capable of enduring. And thus—on the principle sometimes employed in schools, of stimulating by competition and the desire of excelling, or perhaps the fear of being excelled, to an extraordinary exertion on the part of the child, which is afterwards regarded as what he is capable of achieving—the hours of labor and the velocity of the processes were increased to an extent which outraged alike the laws of humanity and of nature.

These evils were observed with concern by many humane and benevolent individuals; and the reflection was frequently thrown out, and with justice, that, whilst we were directing our attention, and expending our ample resources, in affording asylums for the persecuted, and in advocating the cause of freedom abroad, we were culpably neglectful of the system which was being pursued in our manufactories at home. Mr. Wing, in the work before alluded to, who has conferred so signal a benefit on the cause of humanity, by bringing before the public, in an accessible form, the parliamentary evidence on the subject, has observed—"The first cry of the children was at that time (1796) unheeded, owing partly to the magnitude of the events which absorbed the public mind, partly to that apathy with regard to minor sufferings which the contemplation of great atrocities is apt to create. Pity and terror had been too much exhausted by the atrocities of the French Revolution to be easily excited; and the appeals that were then made, not to the legislature, but to the humanity of the public, were made in vain. The evils of the system went on, increasing rather than diminishing, till, at length, the children met with a champion whence it was at least expected. Sir Robert Peel, himself a manufacturer, took up the question in 1802, and brought in a bill, which became an act of Parliament, for the preservation of the health and morals of the apprentices and others employed in cotton and other mills, cotton and other factories. The provisions of the act were—

"1. For the due ventilation and washing of the factories. 2. The proper clothing of the apprentices. 3. The limitation of their labor to *twelve hours daily, and not permitting it at night.* 4. Requiring each apprentice to be instructed, in some part of every working day, during the first four years of his apprenticeship, in reading, writing, and arithmetic. 5. The separation of the sexes. 6. Sunday instruction, and the attendance of the apprentices at divine service, and occasional examination by the rector, vicar, and curate of the parish. 7. Authorizing the justices at quarter sessions to appoint visitors of such factories, with requisite powers."

The extreme cruelty of the existing state of things will appear from the fact, that an act of legislation was necessary to restrict the hours of labor to twelve. Such a *limitation*, it might be expected, would not be objected to by the most avaricious of the proprietors. It was not long, however, before expedients were sought by which this act, moderate as it was in its principles and requirements, might be evaded, or rendered inoperative. The splendid improvements in the construction of the steam-engine, introduced by Watt, and its perfect adaptation to the propulsion of the machinery employed in the factories, furnished the masters with a ready means of escape from the provisions of the act. Rendered independent of the stream as a motive power, the proprietor was now no longer restricted to a particular locality, but might, with equal facility, and even greater advantage, erect his mill in the midst of a populous town. The employment of children residing with their parents in the immediate neighborhood, by relieving him of the trouble and inconvenience connected with a system of apprenticeship, was infinitely preferable. Thus a new and highly advantageous mode of supply was presented, and the system of apprenticeship gradually became extinct. As the act had been framed for the relief of the apprentices, it was, by this unexpected and unforeseen change rendered useless. It soon became necessary, as might have been anticipated, to legislate again upon the subject; for, if an act of protection and limitation was necessary in the former case, it was not less so now.

It was not now a question whether children, at an age at which labor can be borne, and is even desirable in assisting the development and maturity of the frame, can or cannot bear so many hours' labor, but whether children at that tender age, when all the parts of the animal structure are still in an immature and feeble state, before the fibre has attained its full volume and elasticity, before the bones are fully formed, and whilst the growth is proceeding rapidly, and the system, consequently cannot possess that vigor and robustness which are necessary to qualify it for severe and protracted labor—shall or shall not be employed to an almost equal extent. It was not a question whether children, who had received protection and support, with at least a rudimentary education, in some of those institutions in which our country is happily so rich, should be now removed to the scene of their future occupation, but whether those who have not enjoyed those advantages should be, at an early age, deprived of all opportunity of mental and moral cultivation and discipline. It is unnecessary to the present inquiry to detail minutely the successive acts, with their repeals and amendments, which have been made upon this subject. The history is briefly, and in substance, as follows :—

In 1815, a committee was appointed to inquire into the subject, on the motion of Sir Robert Peel, which led to the passing of an act by which it was unlawful to employ a child under nine years of age in a cotton factory, or to allow a young person, under sixteen, to work more than twelve hours a-day. From this moment, then, the subject becomes interesting in a physiological point of view. The question naturally arises, how shall the age which, under the present system of registration, is not unfrequently a matter of considerable uncertainty among the lower classes, be determined, and fraud in this matter be detected? This is a difficulty which, having presented itself at this point, has not since been removed. It is one, however, of very considerable importance in any act of legislation upon the subject, and which it is highly desirable should be thoroughly investigated. For, should the ten-hour bill, which would restrict the labor of both adults and children to these limits, ultimately pass into a law, the temptation will still remain to employ children at too early an age, and the same facilities will exist for practising a deception which shall elude one of the principal objects of legislation, and which will continue to be, as it has been, connived at by both masters and parents.

This act, however, after various alterations and amendments, and which extended only to the cotton factories, was repealed by that bearing the name of Sir John Hobhouse, which made it unlawful to employ any young person under eighteen years of age more than sixty-nine hours a-week.

After a considerable lapse of time, the subject was again agitated, the existing enactments being found insufficient; and Mr. Sadler obtained leave to bring in a bill early in the year 1832. After much discussion, and the production of a great body of evidence, from the clergy, medical men, the operatives themselves, and others, it failed to produce any result. In the following year, Lord Ashley again took up the subject. The bill, however, which his lordship introduced, after much discussion, and the rejection of its second clause in a committee of the house upon it, he was at length induced to relinquish, on the understanding that a government measure would be prepared.

A government measure was at length brought in, the leading features of which were, first, the application of Sir John Hobhouse's act to all mills and factories, with the exception of silk mills; the restriction of the hours of labor for children under thirteen years of age to eight hours a-day, and of young persons under eighteen to sixty-nine hours per week; and, lastly, a provision for education.

Such then, is briefly the history of the legislative measures with respect to the children employed in factory labor.* The sketch which has been given was necessary, to show the deep interest and importance attaching to anything which may be regarded as a criterion of age, inasmuch as the entire efficiency of one principal object of the existing legal enactments depends upon it.

Sir Robert Peel, in his evidence before the committee of 1816, when urging the necessity of either extending his former act, referring to apprentices, to other children, or of some act on their behalf, observes—"Children of the tender age of seven years, and, in some cases, still younger, are frequently admitted to work for thirteen and fourteen hours a-day." The evidence of medical men is scarcely necessary to point out the distressing and almost fatal effects of such a system. The accounts which are given by the children themselves, by their

* For a more circumstantial and particular account of their actual condition and treatment, as well as of the legislative interference in their behalf, the reader is referred to the work already alluded to.

senior companions in toil, or by the overlookers in some instances, are themselves sufficiently eloquent appeals to the sympathy of every humane and benevolent mind. One asks, in an imploring and anxious tone, "Are the hours to be shortened?" The frequent testimony of their fellow-workers is, "they are so tired, that they are unable to sit down or rise up; so tired, that they cannot raise their hands to their heads." Swellings of the feet, knees, and ankles, with curvatures of the spinal column, and other distressing symptoms, were the natural and uniform consequences of such treatment. These evils, however, were not unprovided against by the legislature, for their acts, humanely intentioned and wisely framed as they were, were directed to this very end; but so long as no definite criterion by which the age of the child might be ascertained was given, they were, so far as the objects of their protection were concerned, rendered practically inoperative. It appears, from the first report of the commissioners appointed to inquire into the condition of the factory children, that, "in some instances, children are employed in factories at five years of age—that it is not uncommon to find them there at six, many are under seven, still more under eight but the greater number are there at nine." And, according to the evidence given by the operatives on this occasion, "the weavers are in general idle the early part of the week, and they afterwards work from eighteen to twenty hours to make up their lost time, during which the draw-boy or draw-girl must attend them. I have known, says one, frequent instances of their commencing work at two and three o'clock in the morning.

Such a state of things calls loudly for redress, and claims the earliest attention of the political economist, on account of the physical deterioration which it must produce on that large class of the community inhabiting the manufacturing districts—a consideration of the first importance, when viewed in its connexion and consequences, not only with reference to the existing generation, but as being the probable sources of congenital deformity and imperfect organization in others. On this subject the report of the commissioner states—"The ascertained effects are, in many cases, deformity; in still more, stunted growth, relaxed muscles, and slender conformation ; twisting of the ends of the long bones, relaxation of the ligaments of the knees, ankles, and the like." When such effects are *ascertained* to be produced generally on previously healthy and well-formed children, a considerable probability, at least, is to be apprehended, that similar congenital morbid tendencies will be propagated. It is not, however, the physical deterioration alone, and for its own sake, which is to be deplored, but the evils of still greater moment with which this is almost universally associated. The excessive hurry and toil, and the exhaustion attendant upon the incessant and laborious nature of their occupation, preclude the very attempt at education and the cultivation of the mind ; and the proportion, consequently, of adult operatives who can read or write is exceedingly small. This, too, was an evil which it was attempted to remove, by enacting that children under nine years of age should not be employed in factory labor. A period was thus contemplated sufficient not only for the establishment of health, and the accumulation of natural strength, but also for the acquisition of at least a rudimentary education.

We shall now see in what way these intentions were frustrated, and the provisions of the act evaded. From the great demand for children in factory labor, they have been regarded almost in the light of a species of property; and a man in the manufacturing districts was consequently looked upon as a fortunate individual, who should be possessed of a large family. A great temptation was thus presented to the parents, (with whom it appears most probable that the fraud originated,) to produce false certificates of age. This, on the part of the agents or overlookers, was frequently connived at; and interference, or even detection, on the part of the masters, was hardly to be expected. That such deceptions might, within certain limits, pass unobserved, is not surprising ; they have, however, in some instances, assumed such a formidable aspect, have been "so gross and palpable," as clearly to prove that a mutual understanding on the subject existed among all parties. "I have known cases where the fathers have directed the children to swear that they were upwards of twenty-one years of age, and, before the week was over, it has been discovered, and they have admitted, that they have not turned sixteen. I know of one instance where the parent applied for work for her child at a factory in this town, (Manchester,) when she stated the age to be under fifteen; of course she was refused for night-work. Within a few months, that child was certified, at another factory, to be turned twenty-one, and was taken into employment."—Evidence of Mr. W. Foulkes, solicitor, Manchester.

So constantly were these frauds practised under Sir John Hobhouse's Act, that the committee of inquiry were induced to recommend that some collateral evidence should be sought, and that the certificates should be given by medical men; and, in order to prevent the possibility of any undue influence on the minds of the medical men, by which they might be induced to favor the parents, on whom their practice might, to a considerable extent, depend, these certificates were not to be given by practitioners in the immediate neighborhood, but by men regularly appointed for the purpose. Thus the average power, health, and general appearance of a child of the age the certificated purports to be, would secure, it was considered, the main object—viz. physical qualification for labor. It will be easily seen, however, that a great variety of opinion would be encountered in determining the average power, health, and general appearance and size of a child at any given age; and that a still greater difficulty would present itself in the practical application of these principles, when ascertained. It may also be questioned, whether the apparent physical qualification be all that is required in such cases. Varieties in the development and maturity of the frame present themselves in different individuals; and it is not unfrequently found, that, of two children of the same age, one shall exhibit physical energy and power far greater than the other,—shall exceed him considerably in corporal dimensions, size, stature and weight; it is not, however, it is conceived, to be inferred from these appearances, that he is therefore proportionably advanced in the completion of the formative processes. And if this be not the case, such collateral evidence does not possess all the value and importance which have been attached to it. To indulge in an illustration: to draw such an inference from these appearances, would be as irrational as to expect that the foal of a powerful draught-horse would be sooner fit for labor than that of a race-horse, because it exceeds the latter in absolute weight and bulk. If, on the other hand, it be granted, as it is conceived it readily will be, that the individuals of the human species obey the general law of nature—that is, become developed according to a certain order and within a certain period—then it will immediately be seen of how much greater importance it becomes to ascertain the age than to rely upon physical appearances. Whether this view of the physiology of the matter be correct or not, other difficulties suggested themselves, in practice, of sufficient number and moment to call for some more certain and accessible test of capability. And it was at length decided that the height appeared to afford the desideratum. Mr. Horner, the inspector of the northern division, who was at considerable pains to facilitate the practical working of the Factories' Regulation Acts, after some communications and experimental consideration of the subject, came to the conclusion that the height, with the ordinary strength and appearance, might be regarded as a tolerably certain criterion. And he accordingly gives directions to the surgeons granting certificates, to the effect that more reliance is to be placed upon these combined evidences than upon any certificate of age that could be produced. His directions are—"If you find a child whom you know with certainty to be not more than twelve years of age, with such an unusual degree of development as to be of the ordinary strength and appearance of thirteen, you will be justified in inserting the word 'thirteen' in the certificate." He then proceeds to give directions with respect to the height of the child, and observes—"Unless in cases of unusual development of muscular strength, no child that, without shoes, measures less than 3 feet, 10 inches, ought to be considered as having the appearance of nine years of age; and no child less than 4 feet, $3\frac{1}{2}$ inches, ought to be considered as having the appearance of thirteen years of age."

The height being now determined, much of the ambiguity which pertained to this subject, as it formerly stood, was removed, and the certificating surgeon was relieved from the responsibility which he had previously sustained. To say nothing, however, of the known variability of the height in children, was it not changing the very spirit and intention of the act, when the actual age, though it should be known with certainty, was directed to be totally disregarded? And if it be allowed to be a principle in physiology, to which there is no exception in the human species, that the various parts of an organized structure become developed and matured according to a certain order, and are adapted to enter on their several functions within a certain period, then it must be considered as an innovation to an equal extent upon the economy and purposes of nature. It must be regarded as an unwarrantable interference with her operations, which cannot be unattended with violence and injury to the processes which are being carried on in the frame. In this instance the average height of the child, both of nine and thirteen years of age, appears to be below the standard. So that the evils which would naturally result from the employment of this test, are immensely aggravated by its being made at too low an estimate. A comparison of the average height, as given by Mr. Horner, Mr. Fielden, and Mr. Cowell, will show how cruel and unjust must have been the application of the standard of the first, according to the testimony of the two latter gentlemen.

The average height of a child nine years of age, as given by

| | Ft. | In. |
|---|---|---|
| Mr. Horner, is . . . | 3 | 10 |
| Mr. Fielden, . . . . | 4 | 1 |
| Mr. Cowell, . . . . | 4 | 0 |

The average height of a child of twelve is, according to

| | Ft. | In. |
|---|---|---|
| Mr. Horner, . . . . | 4 | 2 |
| Mr. Fielden, . . . . | 4 | 5½ |
| Mr. Cowell, . . . . | 4 | 5⅛ |

Were such a test allowed to continue in operation, the Factories' Regulation Act must be rendered practically useless. But even this criterion appears to have been in many instances evaded, and, like every other which has been hitherto employed, to have been made the vehicle of deception. Formerly, when the child was to be judged of by its general appearance, in addition to the age, fraud was not uncommonly effected by substituting an elder child to obtain the certificate for a younger sister or brother. Thus, Sarah being thirteen years of age, would apply for a certificate under the name of a younger sister, Jane, perhaps of ten only, which she would readily obtain for her use. And when the fraud by substitution failed, the dress, and every artificial aid that could be resorted to, were so arranged as to impart a more mature appearance to the child than its years justified. These and similar deceptions were now to be provided against, by ascertaining the height, and making subsequent examinations to compare their certified with their actual stature, and thus determine their identity. The resources of the ingenuity and invention of the parents, however, stimulated as they were by their necessitous condition and the desire of gain, were not yet exhausted; and an expedient was actually devised and adopted by which the *height* of the children should be increased. Mr. Wing observes, "I have recently received a communication from a most respectable authority, and to which I can at any time refer, as to a piece of most deceptive sagacity having been lately displayed by parents in cramming cotton into the stockings of their children, so that a fictitious height of an inch or an inch and a quarter has been obtained." But, apart from these instances of deception, a sufficiently grave objection to the employment of a test, admitting confessedly of so much variation as that of the height of children from eight to fourteen years inclusive, presents itself in the severity, and even cruelty, with which it would operate upon those who may have shot up into a rapid growth about the required age, and who would thus be included in the extended hours of labor at a time when, so far from being capable of increased exertion, they would require additional rest and indulgence.

Under such principles of administration, it is clear that any act of legislation, however well it may be adapted to the necessities of the case, must be, to a great extent, neutralized, and must fall short of the object it was designed to accomplish.*

* M. Quetelet observes: "To show how little advancement has been made in the study of the progressive development of the human frame, if

Yet such is the state of things at the present time, and with the ample experience before us of the constant frustration of previous legislative enactments, principally from this practical difficulty in their application, and having at the same time convincing evidence of the failure of the tests hitherto employed, it becomes a consideration of the first moment, in the framing of any new measure upon the subject, to ascertain how far, or with what degree of accuracy, the age may be ascertained ; and what are the means for its discovery, which shall be at the same time the most accessible and the least liable to fallacy.

Such a criterion, it is believed, will be found in the Development of the Teeth ; and it would appear the more surprising that this subject should not long since have been thoroughly investigated, were it not for the known neglect of this interesting branch of study by the medical profession generally, and the culpable dearth of information respecting it in a vast majority of the medical schools. An acquaintance with the manner of their production, their structure, and the mode and order of their eruption, would certainly not discourage the suggestion; on the contrary, their great density of structure, unique and nearly independent mode of formation, together with their almost total want of sympathy with those constitutional changes and variations in the health in which the more highly organized structures partake, would induce the expectation of a greater uniformity of development of the teeth than of any other parts of the frame. And the idea would receive additional confirmation from the well-known fact, that, amongst the lower animals, by common consent, it is regarded as the most certain and constant of all proofs on the subject that can be obtained ; it is, therefore, not unreasonable to suppose, that, making adequate deductions on account of the interruptions to normality of development which a more artificial mode of existence opposes, the same regularity would obtain in the human species.

The composition of the teeth is harder and more compact it were required to establish the age of an individual by the combined consideration of his physiccal qualities, we should not be able to find any scientific rules to guide our determination, but should be obliged to have recourse to the most unsatisfactory empiricism."—See Evils of the Factory System.

than that of any other structure in the body. On account of their great density, and the large proportion of earthy matter which enters into their composition, they are possessed of but a low degree of vitality. The organization, indeed, of the bony structure of the teeth is so feeble, and exhibits so few of the ordinary signs of vascularity, in a healthy and natural state, that some physiologists, leaving out of their view altogether the pathognomic symptoms and the appearances which they assume when in a morbid condition, have refused to them the right to be considered as organized bodies. The teeth, consequently, do not respond in an equal degree with the more vascular parts to those constantly recurring fluctuations in the state of the health. It cannot be denied that they do, to a certain extent, sympathize with constitutional disturbance; but this, when once compared with what takes place in more highly organized parts, is so trifling as scarcely to merit attention. They acquire, for instance, a yellow tinge under affections of the liver, and the minute vessels permeating their substance are susceptible of a sensibly increased action under the influence of mercury, which, from their low organization, generally results in what is termed caries. Still it has been found that even these and still more acute functional disturbances do not, to any appreciable extent, affect the process of formation or development in these organs. A recent instance of this kind was lately mentioned to the writer by a distinguished surgeon in this metropolis, and connected with one of our principal hospitals, in which mercury had been administered as far as the powers of the constitution would allow, for a considerable period, both internally and endermically, and yet no appreciable effect, either of retardation or acceleration in the development of the teeth, was produced. And there is good reason to believe, from repeated observation, that the secretion of the ossific and other matters which enter into the composition of the teeth is never entirely suspended, under either an exalted or depressed state of the health. "There may be, and frequently is, an unequal and scanty deposition of enamel and the bony substance of the tooth may be delicate, and the tooth may consequently be predisposed to those morbid affections to which those organs are liable; but the formative process is all the while in operation—it is never entirely suspended. The growth may be checked, the signs of puberty may be retarded, or appear prematurely, but the teeth, as if belonging to another and separate economy, are uninfluenced by any of these modifying circumstances, at least to anything like the same extent, and become developed according to a law of their own. We frequently see children, who are the subjects of rickets, or other congenital morbid influences, by which the general growth has been retarded, having a very protuberant mouth, and teeth apparently too large for the maxilla; this will be found, however, on a closer inspection, to arise, not from the unusual size of the teeth, nor from their premature development, but from a want of normal growth or expansion of the containing or contiguous parts; the growth of the latter has been retarded, while that of the former has proceeded with its usual regularity."* It would be irrelevant, in this place, to enter into a minute account and description of the manner in which the teeth are produced. To those, however, who are familiar with this process, this circumstance will not appear at all surprising. It will readily be understood, that the same condition of things which would render a part less capable of recovery or rectification, when diseased, will also render it less liable to come into a morbid state. In proportion as the structure is dense, and the vitality low, will an organ possess less sympathy with the fluctuations of the health and constitutional disturbance, and will, consequently, be less liable to have its functions interfered with by morbid influences. The substance of which the teeth are composed, then, being the most dense structure in the frame, may be presumed to be less affected from those causes than any other part. And this may afford a strong *a priori* argument in favor of the teeth as a test where uniformity and constancy of development are required. And it is an opinion which observation and experience tend only to confirm and establish.

The argument which may be drawn from an analogy with the lower animals, will be found still further to corroborate such

* Extract from the Author's Lectures on the Anatomy, Physiology, and Pathology of the Teeth, delivered, during the present session, at St. Thomas's Hospital.

a view. It is difficult to conceive any valid objection to the application of such a test, in this case, as in that of the lower animals. If it be urged that the differences in the mode of life shall produce such changes as to render what may be uniform and constant in the one, variable and fluctuating in the other, it may be urged in reply, that this argument will admit of a double application; and as it will apply with equal force to both sides of the question, it must necessarily neutralize itself. If, amongst the lower animals, which, in the absence of unnatural excitement and the stimulants which are everywhere presented both to the mind and body amongst the individuals of the human species, the teeth have been by common consent preferred to every other criterion of age, it is difficult to conceive why it should not also be the most eligible in the latter case. The effects of this unnatural and artificial mode of existence, as it is sometimes called, operate certainly to the same extent upon the height and general appearance as upon the development and growth of the teeth. And the same regular and quiet mode of life which renders the development of the teeth the most certain criterion of age amongst inferior animals, will also tend, at least in an equal degree, to produce normality of growth, and the general physical appearances. Yet, in the latter case, the height, and general appearance and strength, are never for one moment allowed to come into comparison with the teeth as a criterion of age; and why should it not be so with respect to the human species? It certainly possesses one recommendation, and by no means an inconsiderable one—viz. that it places fraud and deception almost beyond the verge of possibility; and, therefore, should it afford no greater accuracy or certainty than the tests hitherto in use, it would demand consideration and attention, and must be regarded, on this account, as the most valuable criterion which has yet been offered.

It can scarcely be supposed that, by any effort of misdirected ingenuity, the appearances of maturity in the mouth, and its appendages, can by any artifice be simulated. The development and growth of the teeth admit neither of retardation nor acceleration to any perceivable extent at least, by any artificial means; and their general forms and characters are so well defined, that all attempts at deception in any other way are out of the question. It may be urged, that deceptions of this kind are not unfrequently perpetrated upon the teeth of the horse, and that the appearances of another age are simulated almost beyond the power of detection. This, however, is easily explained. It is true, that, in both cases, it is the succession of the second set of teeth (which, in all animals, appear in pairs at regular intervals) which constitutes the test; but as this process is completed at a comparatively early period of the animal's life, an additional circumstance has been taken advantage of, as affording the means of ascertaining the age after the last of the second set of teeth has made its appearance. This arises from the form of the tooth, which presents the appearance of an oval excavation extending a considerable distance upwards from the cutting edge. This fissure becomes filled with a kind of tartar, analogous to that which accumulates on the human teeth, and on the teeth of all animals to a greater or less extent, which, being of a dark color, as contrasted with the surrounding bone, constitutes what is technically called "the mark." For a certain time after they enter upon their functions, these teeth continue to grow, not in their circumference—for, from the manner in which the teeth are formed, and the density of their structure, any subsequent enlargement or growth after they have made their appearance through the gums is impossible—but in the direction of their length, new particles being added at the extremity inclosed within the socket. In proportion as the tooth becomes worn down in mastication, it emerges from the socket, by the deposition of new matter at the opposite extremity; and being in its entire length the arc of a circle, it thus always preserves the same relation to its antagonist. As the fissure, then, before described as existing in the cutting edge of the tooth, extends only to a certain depth, becoming narrower and smaller until it altogether ceases, it is obvious that, as it wears down, this fissure decreases in size, until it altogether disappears, when the abrasion has proceeded so far as to expose that part of the tooth which is beyond it, and which consequently presents a solid and continuous surface. This process, then, which is technically called the "filling up" of the "mark."

is, in reality, nothing more than an effect of the attrition and abrasion of the teeth upon each other. From the uniform nature of the animal's food, and from the regularity of the occurrence of his feeding times, this abrasion and wearing down of the teeth by mastication proceeds in a certain ratio; and thus, by the obliteration of the "mark" occurring within a given time from the appearance of the tooth, an additional evidence is obtained of the age of the animal, after the succession of the second set has failed to be an indication. Now the only way in which deception is practicable is by cauterizing the bone at the cutting edge of the tooth, when this mark has naturally disappeared, which may be so successfully performed as to deceive an unpractised eye. The reminiscence, as it may be termed, of the canine tooth of carnivorous animals, which receives the name of the "tush" in the horse, but which is generally wanting altogether in the mare, is also useful in giving a collateral evidence in forming a judgment of age. And it accordingly is not omitted in deceptions of this kind. This tooth is of a conical form, convex externally, and slightly concave in the direction of its length internally. As it becomes worn down, it of course loses its original form. In order, therefore, that nothing shall be wanting to render the deception complete, this tooth, by the aid of the file and cutting instruments, is made to re-assume, as near as may be, its primitive configuration. This tush, however, which is originally placed at a considerable interval from the front teeth, gradually approaches them; and this circumstance, as it is beyond the reach of mechanical interference, is regarded by many persons as an additional and less fallible collateral evidence on the point.

But however perfect and skilfully performed these frauds may be, it is scarcely credible that they should pass undetected by any one acquainted with the structure and original forms of the teeth. It will immediately be seen, however, that from the simplicity of their forms, their being arranged in an even series, the smallness of their size, and from a variety of circumstances, it is utterly impossible that any deception could be practised upon the human teeth. And even in the case referred to, that of the horse, the frauds are invariably perpetrated in the contrary direction, that is, they are simulations of a younger age. And this is the only way in which it is possible for them to operate; if even it were desired to impose the premature appearance of age, the attempt would be utterly vain; the marks could never be obliterated in less than the usual period, nor could the tush be made to assume the obtuse and rounded form which years alone can impart. Thus, then, it appears utterly impossible, from the very nature of things, that fraud can be practised with respect to the human teeth, much less one which would simulate the premature appearance of age.

It is not contended, however, that the teeth are to be regarded as affording equally accurate indications of physical strength. All that it has been attempted to show is, that their development affords the best possible criterion of age; and age having been, upon the unanimous testimony of the eminent medical gentlemen examined upon the subject, the point of greatest importance in legislation, it must be the most valuable auxiliary that can be obtained in assisting the government in their endeavors for the amelioration of the condition of factory children. It is scarcely necessary to observe, that this test is never to be strictly followed in opposition to certain knowledge of physical incapability. The constitution, state of the health, temperament, &c., will still have to be considered and taken into the account by the certificating surgeon, and serve as so many auxiliaries in deciding upon the physical qualification for labor. But these considerations would be equally taken into the calculation of the certifier were the age actually and incontrovertibly known by certificate. This test, then, is not the less valuable because it is not to be relied on as an indication of natural strength and capability of labor, since the same objection might be urged with equal force in opposition to the certain knowledge which would be afforded by the most perfect system of registration that could be devised.

It is as unnecessary as it would be fruitless, to seek for authorities upon this subject; mere opinions, indeed, obtained from whatever source, unless based upon a tolerably extensive collection of facts, would be of comparatively little value. The present general neglect of the study of dental physiology, its recent and slow extrication from the hands of empirics, and the comparatively late date at which it has become recognised as a distinct branch of medical practice, have all contributed to prevent its receiving its due share of attention, and assuming that rank and station to which its important relations to the system entitle it. It may be well, however, to allude to a parallel case—viz. medico-legal inquiries, in which a certain criterion of age, from the important consequences which it involves, becomes invaluable. Upon this subject, after quoting the law of this country respecting capital and other crimes committed by "infants," Dr. A. T. Thomson has observed, in his admirable lectures on Medical Jurisprudence*—"From this extract from Blackstone, gentlemen, you will perceive, that boys under ten years of age have been tried, condemned, and executed; but that seven years of age is in this country the limit within which crime cannot be legally imputed to any one; there is, however, a precedent of a pardon having been granted to a child who was under seven, who was indicted for homicide; but this case ought not to have gone to a jury, for, as I have already said, the law presumes that, under *seven* years of age, no person can have sufficient discretion, and 'no averment,' says Lord Hale, 'can be received against that 'presumption?' Even under twelve there must be proof of apparent capacity; now, this being the case, it becomes a matter of some consequence to ascertain accurately the age of a precocious child, who is stated to be under *seven* years of age, in an arraingment for felony. How is this fact of absolute infancy to be ascertained? The law says, 'If there be any doubt, the culprit must be brought into court, to be inspected by the judges; and if any doubt still remain, the court may take steps to prove the fact.' The only step that can be taken is, the opinion of a medical man; and in such a case, the practitioner is called upon, not merely to give his opinion, but to state the foundation of it. This is not to be grounded, as you are already aware, upon the development of the intellect; the precocity of mind being influenced by many circumstances, it must rest upon more unvarying evidence;—and this is readily found in the

* Lect. 7, as reported in the Lancet, No. 9, vol. i. 1836–7.

*development of the teeth.* Although this process is occasionally varied in its early stage, yet it is generally regular after the protusion of the temporary or milk teeth." If corroborative opinions were desired, such a decision, so unequivocally expressed, and coming from so distinguished an authority, in a case, too, in which the life or death of the individual depends upon the evidence derived from this source, needs only to be referred to. If, in a case in which the dearest interests of humanity hang upon the decision, where an ignominious and premature death would be the tremendous consequence of an error in judgment, this test, upon mature deliberation, combined with extensive experience and high professional attainments, is pronounced the least fallible, and in every respect the most eligible, it cannot be unworthy the attention of the legislature, in its humane endeavors to succor and protect 35,000 little sufferers from injustice and oppression. In the lecture before alluded to, the indications are pointed out from which a judgment is to be formed respecting the age of the child. "In criminal cases, when nonage is pleaded in extenuation of crime, and no direct proof of age given in evidence, should you be called into court to determine the question, if the third molar tooth have not protruded, you can have *no hesitation* in affirming that the culprit has not passed his seventh year. The same fact may also be taken advantage of, to determine age in some cases of suspected murder. Thus, if a murder has been committed on a boy of *nine* years of age, and, some years after, the skeleton of a boy be found in digging near the spot, however the presumptive evidence of the murder may be thus strengthened by such a circumstance, yet if the third molar tooth be still in the jaw, or rather have not protruded, this skeleton cannot be received in proof of the murder, as the person to whom it belonged could not to have passed his seventh year. Such is the simple method of determining, by a physiological fact, the responsibility of individuals accused of capital crimes in early life." It need scarcely be observed, that, although one age only, seven years, is here alluded to, it is quite sufficient to establish the principle; if the age may be determined at the commencement of the evolution of the second set of teeth, it may be equally practicable in any period during that process.

The second, or permanent, set of teeth begins to make its appearance at seven years of age. The first of these is an additional molar tooth beyond the two on each side of the mouth, in both the upper and under row. The development of this third molar tooth, which constitutes the first of that class of the second set, is the indication on which Dr. Thomson relies with so much confidence. These teeth, as might be expected, do not make their appearance simultaneously; occasionally, one of them begins to be developed at six years, and more frequently at six years and a half; it may be relied on, however, as an almost infallible sign of the child's being seven years of age, if the whole of them have been evolved from the gum. About this period, or soon after, the front teeth, (central incisors,) are cast off, and succeeded by the corresponding teeth of the second set; this takes place in the following order; first, the two central incisors of the lower, then the antagonist teeth in the upper row; the two lateral teeth of the same class in the lower row, followed by a similar succession in the upper. Then omitting the next in the series, the first molar of the temporary set is succeeded by the first bicuspis, or small grinder, of the permanent set. A similar change occurs next in the second molar of the primary set, which is succeeded by the second bicuspis of the permanent set. The cuspidatus, or what is commonly understood as the eye tooth, now begins to emerge from the gum; and these are succeeded by the second molar of the permanent set. This process, occupying the septenary period, ranging from seven to fourteen years of age, occurs normally in the following order:—

| | Years. | |
|---|---|---|
| First molar, or large grinder, . . . . | 7 | |
| Central incisor, or front tooth, . . | 8 | |
| Lateral incisor, . . . . . . . . | 9 | |
| First bicuspis, or small grinder, . . | 10 | |
| Second bicuspis, . . . . . . | 11 | |
| Cuspidatus, or eye tooth, . . . | 12 | 12½ |
| Second molar, or large grinder, . . . | 12½ | 14 |

The second set of teeth, then, consisting of four classes—incisor, canine or cuspid, bicuspid, and molar, deing developed in the order stated above, the mouth will present the following appearances during the entire process:—

| AGE. | Incisor. | | Cuspid. | Bicuspid. | | Molar. | |
|---|---|---|---|---|---|---|---|
| | Central. | Lateral. | | Anterior. | Posterior. | Anterior. | Posterior. |
| Seven years, . : | | | | | | 4 | |
| Eight, . . | 4 | | | | | 4 | |
| Nine, . . . | 4 | 4 | | | | 4 | |
| Ten, . . | 4 | 4 | | 4 | | 4 | |
| Eleven, . . | 4 | 4 | | 4 | 4 | 4 | |
| Twelve to twelve and a half, . | 4 | 4 | 4 | 4 | 4 | 4 | |
| Twelve and a half to fourteen, . | 4 | 4 | 4 | 4 | 4 | 4 | 4 |

This second or permanent set, then, contains an additional eight teeth, making in all twenty-eight. They differ, however, so greatly from those of the first or temporary set, both in size and configuration, that no confusion is likely to arise in determining, during any stage of the process, to which set they belong.

A remarkable instance recently occurred in the private practice of the author, which he is induced to quote, as exhibiting, in a very striking light, the value of evidence founded on these principles. A little girl, apparently not more than five years of age, and who, from a consideration of her physical qualities, height, and general appearance, could not have been pronounced to be six, presented, on an inspection of the mouth, the following appearances: not only were the third molar teeth developed in every direction, but the central and lateral incisors of the second set had made their appearance; the teeth, in fact, irresistibly led to the conclusion, which was not supported by any other sign, that this child was nearly nine instead of five years of age; on inquiry, this was found to be the case—she had nearly completed her ninth year. By a singular coincidence, another young lady, from the same school, having all the appearance of being at least eighteen years of age, on examination, presented every indication of being scarcely fourteen. The cuspidati were not fully developed, nor were the second molar teeth entirely protruded; she was, to the astonishment of all who saw her, under fourteen years of age. The striking contrast which these two extreme cases presented, occurring as they did, at the same time and in the same establishment, will be, it is hoped, a sufficient apology for their introduction in this place; they are, however, only a specimen of a variety of such instances which might be mentioned, and which must continually present themselves to every practitioner.

It may be objected to the introduction of such a test into general use that an extensive and intimate acquaintance with the subject is necessary to qualify an individual to ascertain the age with sufficient accuracy, by the indications thus exhibited; and that, although the development of the teeth may form an exceedingly valuable criterion of the age when rightly understood and accurately applied, yet, in the hands of unprofessional persons, or of those who are not familiar with the subject, it will be unintelligible and fallacious. This is, however, anticipating a difficulty which has no real existence: the fact is, that, although, on a first glance at the subject, it may appear probable that some confusion will occur in this matter, and that it will necessarily be attended with much more complication and perplexity than the former tests, it is infinitely more simple and easy of application than any which could be adopted. From their great uniformity of configuration, size, and arrangement, the teeth possess characters more definite and constant than any other parts; and their physiological history is consequently more precise, and subject to less variation, than that of any other organ. Thus, were it required to determine the age at any period between the ages of seven and fourteen years, (the time occupied in changing the teeth and acquiring the second set, with the exception of the dentes sapientiæ, which require the next septenary period for their evolution,) by preparing consecutive tables, exhibiting a view of the alterations which occur in the mouth extending through that space of time, the utmost facility would be afforded for securing accuracy and expedition. A very trifling degree of industrious application to the subject would enable an individual to distinguish readily between the teeth of the first and those of the second dentition, and between the different classes of each set; their several peculiar characteristics are so well defined, and stand out in such strong relief when contrasted with one another, that the diagnostic features of each, when once recognised, can scarcely be forgotten. These distinctions, then, being borne in mind, and the tables before referred to being carefully prepared, (which would also be extremely valuable in numberless other cases, as of criminal prosecutions, &c., and a variety of circumstances under which it becomes of importance to ascertain the age in the absence or independently of registration,) all that would be necessary would be, to make an inspection of the mouth, and to compare the result with the table, when the age will appear opposite the line containing that number and classes of teeth. Thus, for instance, supposing that the age of a child is required, whose mouth, on inspection, is found to contain twenty-four teeth—viz. four incisors, two canines, and six molars above and below; then, on a reference to the table, if it should be found there stated that a child of eight years of age will have that number of teeth, eight years will be known to be the age of the child; and, again, supposing the mouth be found to contain twenty-eight teeth fully developed, if a statement should be found in the table to the effect that a child of fourteen years of age will have twenty-eight teeth pretty freely developed, no difficulty will exist in predicting the age of the child: the same mode of proceeding will be equally applicable to every intermediate period. These principles, then, being once ascertained, and a sufficient number of facts and observations having been once carefully collated, and digested into accurately prepared tables, the process becomes exeeedingly simple and available—much more so, in fact, because less vague and variable, than the height, general strength and appearance, or the combined consideration of both. And all the complexity of the principle, and arduous study supposed to be necessary to qualify an individual for its successful application, resolve themselves into a system of the utmost perspicuity and simplicity, and a mere acquaintance with the varieties of form which the different classes of the teeth assume. If, however, only two epochs—viz. nine and thirteen years of age—will be, as hitherto, required to be ascertained for the successful operation of the Factories Regulation Act, the process will be still further simplified.

Acting on the supposition that these periods only will be sought, and these being amply sufficient for the resolution of the question proposed at the commencement of this inquiry; the writer has confined himself in his present investigations to these two legislative epochs. The results of these investigations he has appended to this paper, and on them, more than on any arguments, however convincing they may appear, or, however difficult it may seem to escape from the conclusions to which they lead, does he rely for an assent to his opinions or credit to his deductions, well knowing that to these only can he appeal for a decision, and that they alone can confer value and the weight of authority to the views he has entertained on the subject. The inquiry was undertaken from a conviction of the necessity of some certain and well-defined principles of estimating the age in children, in consequence of the failure of all the criteria hitherto in use, as well as from a consideration of the great importance which a well-established test of this kind would possess in facilitating the operation of any legislative enactments upon this subject; and it has been conducted with the strictest regard to fairness in the selection of the individuals who were submitted to observation, and of accuracy of detail in the appearances they presented. It was originally intended, in order to arrive as nearly as possible at accuracy of observation, to record the exact age in years and months, and to state the temperament, general aspect of the child, and whether it had been the subject of the ordinary catalogue of infantile diseases. It was subsequently found, however, that these particulars afforded no certain data on which to account for the deviations which sometimes presented themselves; and as they necessarily tended to embarrass the tables, and to render them too minute and complicated to be practically valuable, they have been since disregarded. In the course of the inquiry, upwards of 1000 children have been examined from various classes in society; in the majority of instances, however, the investigation has been conducted on those from the lower classes, as being better adapted to the present purpose. The examinations were instituted in the order in which the tables appear, the inmates of the Blue-Coat School, Westminster, were first subjected to observation, and the rest in succession as they are arranged in manner following:—

Sixteen boys, about the age of nine years,* that were examined in the Blue-Coat School, Westminster, presented the following appearances :—

| BOYS OF NINE YEARS OF AGE. | Incisor. Central. | Incisor. Lateral. | Cuspid. | Bicuspid. Anterior. | Bicuspid. Posterior. | Molar. Anterior. | Molar. Posterior. |
|---|---|---|---|---|---|---|---|
| Two had . . . | 4 | 4 | | | | 4 | |
| Ten . . . . . . | 4 | 3 | | | | 4 | |
| Three . . . . | 4 | | | | | 4 | |
| One . . . . . . | 3 | 2 | | | | 4 | |

There being but few girls of the required age in this establishment, it was thought unnecessary to enter upon their examination. The variations in the appearances of the mouth which these children exhibited are trifling, their differences of height considerable. A more uneven regiment, when ranged together could scarcely have been selected. Judging from their height, indeed, they were of all ages, from six to twelve or thirteen years, while the teeth would not have indicated a range of more than nine months.

Previously to entering upon this inquiry, my investigations had been conducted on children of the middle and upper classes; and forming an estimate from the data thus obtained, the development of the teeth in these boys appeared tardy and imperfect. Whilst pursuing my inquiry, however, I became convinced that the appearances exhibited by the former were somewhat in advance of the latter. A similar remark may be made with respect to the sexes. As a general rule, the development of the teeth in the girls was more regular, and a little in advance of that of the boys.

I next proceeded to an examination of the boys in the Central National School, Westminster. The following is a summary of the appearances presented, of forty boys about nine years of age :—

* There were only two boys in this school of the age of thirteen; they will therefore be classed with some others subsequently examined.

| BOYS OF NINE YEARS OF AGE. | Incisor. Central. | Incisor. Lateral. | Cuspid. | Bicuspid. Anterior. | Bicuspid. Posterior. | Molar. Anterior. | Molar. Posterior. |
|---|---|---|---|---|---|---|---|
| Fifteen had . . . | 4 | 4 | | | | 4 | |
| Fourteen . . . | 4 | 3 | | | | 4 | |
| Eight . . . . . | 4 | 2 | | | | 4 | |
| One . . . . . | 4 | | | | | 4 | |
| Two . . . . . | 3 | 2 | | | | 4 | |

Fifteen of thirteen years of age had—

| BOYS OF THIRTEEN YEARS OF AGE. | Incisor. Central. | Incisor. Lateral. | Cuspid. | Bicuspid. Anterior. | Bicuspid. Posterior. | Molar. Anterior. | Molar. Posterior. |
|---|---|---|---|---|---|---|---|
| Eight had . . . | 4 | 4 | 4 | 4 | 4 | 4 | 4 |
| One . . . . . . | 4 | 4 | 2 | 2 | 2 | 4 | 2 |
| Two . . . . . . | 4 | 4 | 3 | 3 | 3 | 4 | 1 |
| Two . . . . . . | 4 | 4 | 2 | 4 | 2 | 4 | 1 |
| Two . . . . . . | 4 | 4 | 1 | 2 | 2 | 4 | 1 |

In the same establishment there were thirty girls of nine years of age, of whom—

| GIRLS OF NINE YEARS OF AGE. | Incisor. Central. | Incisor. Lateral. | Cuspid. | Bicuspid. Anterior. | Bicuspid. Posterior. | Molar. Anterior. | Molar. Posterior. |
|---|---|---|---|---|---|---|---|
| Nineteen had . . . | 4 | 4 | | | | 4 | |
| Five . . . . . . | 4 | 3 | | | | 4 | |
| Three . . . . . . | 4 | 2 | | | | 4 | |
| Two . . . . . . | 4 | 1 | | | | 4 | |
| One . . . . . . | 4 | | | | | 4 | |

There were four of thirteen years of age—

| GIRLS OF THIRTEEN YEARS OF AGE. | Incisor. Central. | Incisor. Lateral. | Cuspid. | Bicuspid. Anterior. | Bicuspid. Posterior. | Molar. Anterior. | Molar. Posterior. |
|---|---|---|---|---|---|---|---|
| Three had . . . | 4 | 4 | 3 | 4 | 2 | 4 | 3 |
| One . . . . . . | 4 | 4 | 2 | 4 | | 4 | |

In the Foundling Hospital there were eleven boys of the age of nine; of these—

| BOYS OF NINE YEARS OF AGE. | Incisor. Central. | Incisor. Lateral. | Cuspid. | Bicuspid. Anterior. | Bicuspid. Posterior. | Molar. Anterior. | Molar. Posterior. |
|---|---|---|---|---|---|---|---|
| Seven had . . . | 4 | 4 | | | | 4 | |
| One . . . . . . | 4 | 3 | | | | 4 | |
| One . . . . . . | 4 | 2 | | | | 4 | |
| One . . . . . . | 4 | | | | | 4 | |
| One . . . . . . | 3 | | | | | 4 | |

There were in the same establishment fourteen boys of thirteen years of age—

| BOYS OF THIRTEEN YEARS OF AGE. | Incisor. Central. | Incisor. Lateral. | Cuspid. | Bicuspid. Anterior. | Bicuspid. Posterior. | Molar. Anterior. | Molar. Posterior. |
|---|---|---|---|---|---|---|---|
| Nine had . . . | 4 | 4 | 4 | 4 | 4 | 4 | 4 |
| Three . . | 4 | 4 | 3 | 4 | 3 | 4 | 3 |
| Two . . | 4 | 4 | 2 | 3 | 4 | 4 | 2 |

The boys in this establishment were all of low stature: there was, however, great irregularity in this respect, some being considerably shorter than others. Two boys who were standing together, and to whom my attention was directed by the master whilst I was pursuing my examinations, particularly attracted my notice. They exhibited in so striking a manner the superiority of the teeth over the height, as a criterion of age, that I was induced to measure them, and record the stature and the appearances of the mouth in parallel columns. They, however, constitute only one of a number of instances which continually occurred during the progress of this inquiry.

| | HEIGHT. Feet. | HEIGHT. Inches. | AGE. Years. | AGE. Months. | INCISOR. Central. | INCISOR. Lateral. | MOLAR. Ante'r. |
|---|---|---|---|---|---|---|---|
| James Jacques . | 4 | 0¾ | 8 | 4 | 3 | | 4 |
| John Sims . . . | 3 | 7¼ | 8 | 7 | 3 | 2 | 4 |

Here, then, was a difference of *five inches and a half* in height in favor of the youngest boy, who was by *three months* the junior of the other. It need scarcely be observed, that the elder and shorter boy was by far the strongest and the most capable of exertion; his pulse was strong and regular, while that of the taller child was small and frequent, indicating a low degree of animal power. Judging, then, from the height in this case, the elder boy, who was active and shrewd, and displayed great physical and mental energy, would have been pronounced too young for labor, while the other, *who was his junior by three months*, but who exceeded him in stature by *five inches and a half*, would have been subjected to a distressing and injurious amount of exertion. It will be seen, however, that the appearances of the mouth indicated the true state of the case; so that, relying upon these, the shorter child must have been the elder.

There were but five girls in this institution of nine years of age; of these—

| GIRLS OF NINE YEARS OF AGE. | Incisor. Central. | Incisor. Lateral. | Cuspid. | Bicuspid. Ante-rior. | Bicuspid. Poste-rioi. | Molar. Ante-rior. | Molar. Poste-rior. |
|---|---|---|---|---|---|---|---|
| Four had . | 4 | 4 | | | | 4 | |
| One . . | 4 | 3 | | | | 4 | |

There were eight of thirteen years of age, who all presented very nearly the same appearances, with the exception of the second, or posterior, molar.

| GIRLS OF THIRTEEN YEARS OF AGE. | Incisor. Central. | Incisor. Lateral. | Cuspid. | Bicuspid. Ante-rior. | Bicuspid. Poste-rior. | Molar. Ante-rior. | Molar. Poste-rior. |
|---|---|---|---|---|---|---|---|
| Four had . . . | 4 | 4 | 4 | 4 | 4 | 4 | 4 |
| Two . . . . . | 4 | 4 | 4 | 4 | 4 | 4 | 3 |
| Two . . . . . | 4 | 4 | 4 | 4 | 4 | 4 | 2 |

There were fifteen boys in the city of London National School, Doctors' Commons; of these—

| BOYS OF NINE YEARS OF AGE. | Incisor. Central. | Incisor. Lateral. | Cuspid. | Bicuspid. Ante-rior. | Bicuspid. Poste-rior. | Molar. Ante-rior. | Molar. Poste-rior. |
|---|---|---|---|---|---|---|---|
| Six had . . . . | 4 | 4 | | | | 4 | |
| Five . . . . . | 4 | 3 | | | | 4 | |
| Three . . . . | 4 | 2 | | | | 4 | |
| One . . . . . | 4 | | | | | 4 | |

Five of thirteen years of age—

| BOYS OF THIRTEEN YEARS OF AGE. | Incisor. Central. | Incisor. Lateral. | Cuspid. | Bicuspid. Ante-rior. | Bicuspid. Poste-rior. | Molar. Ante-rior. | Molar. Poste-rior. |
|---|---|---|---|---|---|---|---|
| Three* had . | 4 | 4 | 4 | 4 | 4 | 4 | 4 |
| One . . . | 4 | 4 | 4 | 4 | 3 | 4 | 3 |
| One . . . | 4 | 4 | 4 | 4 | 4 | 4 | 1 |

In the same establishment there were were five girls of nine years of age—

| GIRLS OF NINE YEARS OF AGE. | Incisor. Central. | Incisor. Lateral. | Cuspid. | Bicuspid. Ante-rior. | Bicuspid. Poste-rior. | Molar. Ante-rior. | Molar. Poste-rior. |
|---|---|---|---|---|---|---|---|
| One had . . | 4 | 4 | | | | 4 | |
| Two . . . | 4 | 3 | | | | 4 | |
| One . . | 4 | 2 | | | | 4 | |
| One . . . | 4 | 4 | 4 | 4 | 4 | 4 | 3 |

* With an occasional variation in the development of one or two of the bicuspides, or small double teeth, which is immaterial.

This last, having the development of a girl of thirteen, being the only instance of such a deviation, I am induced to suppose in error with respect to her age. I have made inquiries, but, in the absence of any register, nothing certain can be elicited.

There were six of thirteen; of these—

| GIRLS OF THIRTEEN YEARS OF AGE. | Incisor. Central. | Incisor. Lateral. | Cuspid. | Bicuspid. Anterior. | Bicuspid. Posterior. | Molar. Anterior. | Molar. Posterior. |
|---|---|---|---|---|---|---|---|
| Four had . . | 4 | 4 | 4 | 4 | 4 | 4 | 4 |
| One . . . | 4 | 4 | 4 | 4 | 4 | 4 | 3 |
| One . . | 4 | 4 | 3 | 4 | 3 | 4 | 1 |

I proceeded next to the city of London National School, in Shoe-Lane. I there found, of nineteen boys of nine years of age—

| BOYS OF NINE YEARS OF AGE. | Incisor. Central. | Incisor. Lateral. | Cuspid. | Bicuspid. Anterior. | Bicuspid. Posterior. | Molar. Anterior. | Molar. Posterior. |
|---|---|---|---|---|---|---|---|
| Eleven had . . | 4 | 4 | | | | 4 | |
| Four . . | 4 | 3 | | | | 4 | |
| Three . . | 4 | 2 | | | | 4 | |
| One . . . | 1 | 1 | | | | 4 | |

Four of thirteen—

| BOYS OF THIRTEEN YEARS OF AGE. | Incisor. Central. | Incisor. Lateral. | Cuspid. | Bicuspid. Anterior. | Bicuspid. Posterior. | Molar. Anterior. | Molar. Posterior. |
|---|---|---|---|---|---|---|---|
| One had . . | 4 | 4 | 4 | 4 | 3 | 4 | 3 |
| Three . . | 4 | 4 | 2 | 4 | 3 | 4 | 1 |

Of fifteen girls, in the same school, of the age of nine—

| GIRLS OF NINE YEARS OF AGE. | Incisor. Central. | Incisor. Lateral. | Cuspid. | Bicuspid. Anterior. | Bicuspid. Posterior. | Molar. Anterior. | Molar. Posterior. |
|---|---|---|---|---|---|---|---|
| Thirteen had . | 4 | 4 | | | | 4 | |
| Two . . | 4 | 3 | | | | 4 | |

There were three of thirteen years—

| GIRLS OF THIRTEEN YEARS. OF AGE. | Incisor. Central. | Incisor. Lateral. | Cuspid. | Bicuspid. Anterior. | Bicuspid. Posterior. | Molar. Anterior. | Molar. Posterior. |
|---|---|---|---|---|---|---|---|
| Two had . . | 4 | 4 | 4 | 4 | 4 | 4 | 4 |
| One . . | 4 | 4 | 4 | 3 | 4 | 4 | 4 |

The difference of one bicuspid, or small molar tooth, is so trifling, that these three girls may fairly be said to have presented the same developments. For all practical purposes, indeed, wherever the tooth on one side of the mouth is freely developed, it would be quite fair to reckon the two as having emerged from the gum. The teeth being formed in pairs, and *normally* making their appearance nearly at the same time, if one should have acquired its proper degree of evolution, but, from a want of consentaneous action of the absorbents on the temporary tooth, or from some mechanical or other obstruction, its fellow is retained within the gums, *practically* the pair may be considered as present. This principle, however, will only be allowed in cases in which one of the pair is *freely* developed, and must then be restricted to the teeth of each row. Thus, should any pair of teeth be present in the upper, which are absent in the lower row, their existence must not in this way be inferred. There can, however, it is conceived, be no possible objection to the application of such a principle while it is confined to the teeth of each row.

I now proceeded to an examination of the children in the St. Andrews' Charity School, Hatton Garden. It contained fifteen boys of nine years of age; of these—

| BOYS OF NINE YEARS OF AGE. | Incisor. | | | Bicuspid. | | Molar. | |
|---|---|---|---|---|---|---|---|
| | Central. | Lateral. | Cuspid. | Anterior. | Posterior. | Anterior. | Posterior |
| Nine had . | 4 | 4 | | | | 4 | |
| Three . . | 4 | 3 | | | | 4 | |
| One . . | 3 | 3 | | | | 4 | |
| Two . . | 2 | 2 | | | | 4 | |

Eighteen of the age of thirteen—

| BOYS OF THIRTEEN YEARS OF AGE | Incisor. | | | Bicuspid. | | Molar. | |
|---|---|---|---|---|---|---|---|
| | Central. | Lateral. | Cuspid. | Anterior. | Posterior. | Anterior. | Posterior. |
| Thirteen* had . | 4 | 4 | 4 | 4 | 4 | 4 | 4 |
| One . . | 4 | 4 | 3 | 4 | 2 | 4 | 3 |
| Two . . | 4 | 4 | 4 | 4 | 3 | 4 | 2 |
| One . . . | 4 | 4 | 3 | 1 | | 4 | 1 |
| One . . | 4 | 4 | | 2 | | 4 | |

* Two of these, however, were deficient in the canine teeth. I have included them because of the uniformity of the second molar, the most important tooth as an indication at this age.

Of the accuracy of the age of this last there was some doubt.

There were in this school eighteen girls of nine years of age; of these—

| GIRLS OF NINE YEARS OF AGE. | Incisor. | | Cuspid. | Bicuspid. | | Molar. | |
|---|---|---|---|---|---|---|---|
| | Central. | Lateral. | | Anterior. | Posterior. | Anterior. | Posterior. |
| Nine had* . | 4 | 4 | | | | 4 | |
| Five . . | 4 | 3 | | | | 4 | |
| One . . . | 4 | 1 | | | | 4 | |
| One . . | 3 | 3 | | | | 4 | |
| Two . . | 3 | | | | | 4 | |

There were, also, eighteen girls of thirteen years old; of these—

| GIRLS OF THIRTEEN YEARS OF AGE. | Incisor. | | Cuspid. | Bicuspid. | | Molar. | |
|---|---|---|---|---|---|---|---|
| | Central. | Lateral. | | Anterior. | Posterior. | Anterior. | Posterior. |
| Fifteen† had . | 4 | 4 | 4 | 4 | 4 | 4 | 4 |
| One . . | 4 | 4 | 4 | 3 | 4 | 4 | 3 |
| Two . . | 4 | 4 | 3 | 4 | 3 | 4 | 2 |

I now had an opportunity of examining the teeth of the children in the Central National School, White-street, Little Moorfields. I there found thirty-seven boys of nine years of age, presenting the following appearances:—

| BOYS OF NINE YEARS OF AGE. | Incisor. | | Cuspid. | Bicuspid. | | Molar. | |
|---|---|---|---|---|---|---|---|
| | Central. | Lateral. | | Anterior. | Posterior. | Anterior. | Posterior. |
| Eighteen had . | 4 | 4 | | | | 4 | |
| Six . . . | 4 | 3 | | | | 4 | |
| Seven . . . | 4 | 2 | | | | 4 | |
| Two . . | 4 | | | | | 4 | |
| Two . . . | 3 | 2 | | | | 4 | |
| Two . . | 3 | | | | | 4 | |

Seventeen, of the age of thirteen years, had the following developments:—

* In three of these, the cuspid and posterior bicuspid teeth of the lower row were just making their appearance.

† The most important teeth to be discovered at this age are, of course the cuspid and posterior molar; an occasional variation, consequently, of the bicuspides is immaterial. There were three such cases among these.

| BOYS OF THIRTEEN YEARS OF AGE. | Incisor. Central. | Incisor. Lateral. | Cuspid. | Bicuspid. Anterior. | Bicuspid. Posterior. | Molar. Anterior. | Molar. Posterior. |
|---|---|---|---|---|---|---|---|
| Nine had | 4 | 4 | 4 | 4 | 4 | 4 | 4 |
| Three | 4 | 4 | 3 | 4 | 4 | 4 | 3 |
| One | 4 | 4 | 2 | 4 | 4 | 4 | 4 |
| One | 4 | 4 | | 4 | 2 | 4 | 4 |
| Three | 4 | 4 | 3 | 4 | 3 | 4 | 2 |

In the same school there were sixteen girls of nine years of age; of these—

| GIRLS OF NINE YEARS OF AGE. | Incisor. Central. | Incisor. Lateral. | Cuspid. | Bicuspid. Anterior. | Bicuspid. Posterior. | Molar. Anterior. | Molar. Posterior. |
|---|---|---|---|---|---|---|---|
| Eleven had | 4 | 4 | | | | 4 | |
| One | 4 | 3 | | | | 4 | |
| Three | 4 | 2 | | | | 4 | |
| One | 3 | | | | | 4 | |

Six girls of thirteen years old; of these—

| GIRLS OF THIRTEEN YEARS OF AGE. | Incisor. Central. | Incisor. Lateral. | Cuspid. | Bicuspid. Anterior. | Bicuspid. Posterior. | Molar. Anterior. | Molar. Posterior. |
|---|---|---|---|---|---|---|---|
| Four had | 4 | 4 | 4 | 4 | 4 | 4 | 4 |
| One | 4 | 4 | 4 | 4 | 4 | 4 | 3 |
| One | 4 | 4 | | 4 | 4 | 4 | |

In the Bishopsgate National and Parochial Schools there were thirty-four boys of nine years of age; of these—

| BOYS OF NINE YEARS OF AGE. | Incisor. Central. | Incisor. Lateral. | Cuspid. | Bicuspid. Anterior. | Bicuspid. Posterior. | Molar. Anterior. | Molar. Posterior. |
|---|---|---|---|---|---|---|---|
| Thirteen had | 4 | 4 | | | | 4 | |
| Nine | 4 | 3 | | | | 4 | |
| Seven | 4 | 2 | | | | 4 | |
| One | 4 | | | | | 4 | |
| Four | 3 | 3 | | | | 4 | |

Eleven, of the age of thirteen years, had the following developments:—

| BOYS OF THIRTEEN YEARS OF AGE. | Incisor. Central. | Incisor. Lateral. | Cuspid. | Bicuspid. Anterior. | Bicuspid. Posterior. | Molar. Anterior. | Molar. Posterior. |
|---|---|---|---|---|---|---|---|
| Three had | 4 | 4 | 4 | 4 | 4 | 4 | 4 |
| Four | 4 | 4 | 4 | 4 | 4 | 4 | 3 |
| Four | 4 | 4 | 2 | 4 | 3 | 4 | 2 |

In the same establishment there were thirty-four girls, who presented the following appearances :—

| GIRLS OF NINE YEARS OF AGE. | Incisor. Central. | Incisor. Lateral. | Cuspid. | Bicuspid. Anterior. | Bicuspid. Posterior. | Molar. Anterior. | Molar. Posterior. |
|---|---|---|---|---|---|---|---|
| Nineteen* had | 4 | 4 | | | | 4 | |
| Nine | 4 | 3 | | | | 4 | |
| Two | 4 | 2 | | | | 4 | |
| Two | 4 | | | | | 4 | |
| Two | 3 | 3 | | | | 4 | |

Six of thirteen exhibited the following developments:

| GIRLS OF THIRTEEN YEARS OF AGE. | Incisor. Central. | Incisor. Lateral. | Cuspid. | Bicuspid. Anterior. | Bicuspid. Posteiror. | Molar. Anterior. | Molar. Posterior. |
|---|---|---|---|---|---|---|---|
| Four had | 4 | 4 | 4 | 4 | 3 | 4 | 4 |
| One | 4 | 4 | 4 | 4 | 3 | 4 | 3 |
| One | 4 | 4 | 4 | 3 | 3 | 4 | 2 |

Amongst the boys of the National school, Hoxton-square, Old Street Road, I found forty-eight of the age of nine years. Of these—

| BOYS OF NINE YEARS OF AGE. | Incisor. Central. | Incisor. Lateral. | Cuspid. | Bicuspid. Anterior. | Bicuspid. Posterior. | Molar. Anterior. | Molar. Posterior. |
|---|---|---|---|---|---|---|---|
| Seventeen had | 4 | 4 | | | | 4 | |
| Seven | 4 | 3 | | | | 4 | |
| Thirteen | 4 | 2 | | | | 4 | |
| Eight | 4 | | | | | 4 | |
| Two | 3 | 2 | | | | 4 | |
| One | 3 | | | | | 4 | |

Five of thirteen years of age had as follows :

| BOYS OF THIRTEEN YEARS OF AGE. | Incisor. Central. | Incisor. Lateral. | Cuspid. | Bicuspid. Anterior. | Bicuspid. Posterior. | Molar. Anterior. | Molar. Posterior. |
|---|---|---|---|---|---|---|---|
| One had | 4 | 4 | 4 | 4 | 4 | 4 | 4 |
| Two | 4 | 4 | 4 | 4 | 4 | 4 | 3 |
| One | 4 | 4 | 3 | 4 | 3 | 4 | 2 |
| One | 4 | 4 | 3 | 4 | 3 | 4 | 1 |

There were only eleven girls of nine years of age in this institution; of these—

* Two of these had changed their lower cuspid teeth.

| GIRLS OF NINE YEARS OF AGE. | Incisor. Central. | Incisor. Lateral. | Cuspid. | Bicuspid. Anterior. | Bicuspid. Posterior. | Molar. Anterior. | Molar. Posterior. |
|---|---|---|---|---|---|---|---|
| Nine had . . | 4 | 4 | | | | 4 | |
| Two . . . | 4 | 3 | | | | 4 | |

Four of thirteen years of age had the following developments:

| GIRLS OF THIRTEEN YEARS OF AGE. | Incisor. Central. | Incisor. Lateral. | Cuspid. | Bicuspid. Anterior. | Bicuspid. Posterior. | Molar. Anterior. | Molar. Posterior. |
|---|---|---|---|---|---|---|---|
| Two had, . . | 4 | 4 | 4 | 4 | 4 | 4 | 4 |
| One . . . . | 4 | 4 | 4 | 4 | 2 | 4 | 3 |
| One . . . | 4 | 4 | 3 | 4 | 2 | 4 | 2 |

Of twenty-three boys in the Maida-Hill National School, of nine years of age—

| BOYS OF NINE YEARS OF AGE. | Incisor. Central. | Incisor. Lateral. | Cuspid. | Bicuspid. Anterior. | Bicuspid. Posterior. | Molar. Anterior. | Molar. Posterior. |
|---|---|---|---|---|---|---|---|
| Fifteen had* . | 4 | 4 | | | | 4 | |
| Three . . | 4 | 3 | | | | 4 | |
| Four . . . | 4 | 2 | | | | 4 | |
| One . . · | 3 | 1 | | | | 4 | |

Of nine of thirteen years old—

| BOYS OF THIRTEEN YEARS OF AGE. | Incisor. Central. | Incisor. Lateral. | Cuspid. | Bicuspid. Anterior. | Bicuspid. Posterior. | Molar. Anterior. | Molar. Posterior. |
|---|---|---|---|---|---|---|---|
| Five had . . | 4 | 4 | 4 | 4 | 4 | 4 | 4 |
| One . . . . | 4 | 4 | 4 | 4 | 4 | 4 | 3 |
| Two . . . | 4 | 4 | | 3 | 4 | 4 | 4 |
| One . . . | 4 | 4 | | 3 | 2 | 4 | 2 |

In the same establishment there were eighteen girls of nine years old; of these—

| GIRLS OF NINE YEARS OF AGE. | Incisor. Central. | Incisor. Lateral. | Cuspid. | Bicuspid. Anterior. | Bicuspid. Posterior. | Molar. Anterior. | Molar. Posterior. |
|---|---|---|---|---|---|---|---|
| Twelve† had . | 4 | 4 | | | | 4 | |
| Three . . | 4 | 3 | | | | 4 | |
| Three . . . | 4 | 2 | | | | 4 | |

There were only two girls in this institution of thirteen years of age; they will therefore be included in the same table with

* In two of these the cuspid tooth of the lower row was just making its appearance.

† One had a slight development of the cuspid tooth in the lower row.

those of the next school visited, viz. the St. Mary's, or Western National School. There were in this establishment fifty-six boys, at nine years of age, who had the following developments:—

| BOYS OF NINE YEARS OF AGE. | Incisor. Central. | Incisor. Lateral. | Cuspid. | Bicuspid. Anterior. | Bicuspid. Posterior. | Molar. Anterior. | Molar. Posterior. |
|---|---|---|---|---|---|---|---|
| Thirty had . . | 4 | 4 | | | | 4 | |
| Three . . | 4 | 3 | | | | 4 | |
| Fourteen . . | 4 | 2 | | | | 4 | |
| Five . . . | 4 | | | | | 4 | |
| Four . . . | 3 | | | | | 4 | |

Twelve of thirteen years old exhibited the following appearances:—

| BOYS OF THIRTEEN YEARS OF AGE. | Incisor. Central. | Incisor. Lateral. | Cuspid. | Bicuspid. Anterior. | Bicuspid. Posterior. | Molar. Anterior. | Molar. Posterior. |
|---|---|---|---|---|---|---|---|
| Four had . . | 4 | 4 | 4 | 4 | 4 | 4 | 4 |
| Three . . | 4 | 4 | 3 | 4 | 3 | 4 | 3 |
| One . . . | 4 | 4 | 4 | 4 | 4 | 4 | 2 |
| Four . . | 4 | 4 | 3 | 4 | 4 | 4 | 1 |

There were thirty-two-girls of nine years of age; of these—

| GIRLS OF NINE YEARS OF AGE. | Incisor. Central. | Incisor. Lateral. | Cuspid. | Bicuspid. Anterior. | Bicuspid. Posterior. | Molar. Anterior. | Molar. Posterior. |
|---|---|---|---|---|---|---|---|
| Twenty-five had, . | 4 | 4 | | | | 4 | |
| Five . . | 4 | 3 | | | | 4 | |
| One . . . | 4 | 2 | | | | 4 | |
| One . . . | 3 | | | | | 4 | |

Twelve of thirteen years old had the following developments:—

| GIRLS OF THIRTEEN YEARS OF AGE. | Incisor. Central. | Incisor. Lateral. | Cuspid. | Bicuspid. Anterior. | Bicuspid. Posterior. | Molar. Anterior. | Molar. Posterior. |
|---|---|---|---|---|---|---|---|
| Eleven had . . | 4 | 4 | 4 | 4 | 4 | 4 | 4 |
| One had . . | 4 | 4 | 3 | 4 | 3 | 4 | 3 |

Of forty-six boys in the St. James' National School—

| BOYS OF NINE YEARS OF AGE. | Incisor. Central. | Incisor. Lateral. | Cuspid. | Bicuspid. Anterior. | Bicuspid. Posterior. | Molar. Anterior. | Molar. Posterior. |
|---|---|---|---|---|---|---|---|
| Twenty-four had . | 4 | 4 | | | | 4 | |
| Three . . | 4 | 3 | | | | 4 | |
| Eleven . . | 4 | 2 | | | | 4 | |
| Three . . | 4 | | | | | 4 | |
| Three . . . | 3 | 2 | | | | 4 | |
| Two . . | 3 | | | | | 4 | |

There were only five of thirteen years old ; of these—

| BOYS OF THIRTEEN YEARS OF AGE. | Incisor. Central. | Incisor. Lateral. | Cuspid. | Bicuspid. Anterior. | Bicuspid. Posterior. | Molar. Anterior. | Molar. Posterior. |
|---|---|---|---|---|---|---|---|
| Two had . . . | 4 | 4 | 4 | 4 | 4 | 4 | 4 |
| One . . . . | 4 | 4 | 4 | 4 | 3 | 4 | 3 |
| Two . . . . | 4 | 4 | 4 | 4 | 3 | 4 | 1 |

There were in the same establishment thirty-five girls, of nine years of age, who had the following developments :—

| GIRLS OF NINE YEARS OF AGE. | Incisor. Central. | Incisor. Lateral. | Cuspid. | Bicuspid. Anterior. | Bicuspid. Posterior. | Molar. Anterior. | Molar. Posterior. |
|---|---|---|---|---|---|---|---|
| Twenty-three had | 4 | 4 | | | | 4 | |
| Five . . . . | 4 | 3 | | | | 4 | |
| Six . . . . | 4 | 2 | | | | 4 | |
| One . . . . | 3 | 1 | | | | 4 | |

Of seven, of thirteen years of age—

| GIRLS OF THIRTEEN YEARS OF AGE. | Incisor. Central. | Incisor. Lateral. | Cuspid. | Bicuspid. Anterior. | Bicuspid. Posterior. | Molar. Anterior. | Molar. Posterior. |
|---|---|---|---|---|---|---|---|
| Two had . . . | 4 | 4 | 4 | 4 | 4 | 4 | 4 |
| Three . . . | 4 | 4 | 3 | 4 | 3 | 4 | 3 |
| Two . . . . | 4 | 4 | 3 | 4 | 4 | 4 | 1 |

Amongst the girls of the Burlington School, I found thirteen at nine years of age ; of these—

| GIRLS OF NINE YEARS OF AGE. | Incisor. Central. | Incisor. Lateral. | Cuspid. | Bicuspid. Anterior. | Bicuspid. Posterior. | Molar. Anterior. | Molar. Posterior. |
|---|---|---|---|---|---|---|---|
| Nine had . . . | 4 | 4 | | | | 4 | |
| One . . . . | 4 | 3 | | | | 4 | |
| Three . . . | 4 | 2 | | | | 4 | |

And ten of thirteen years of age, who presented the following appearances :—

| GIRLS OF THIRTEEN YEARS OF AGE. | Incisor. Central. | Incisor. Lateral. | Cuspid. | Bicuspid. Anterior. | Bicuspid. Posterior. | Molar. Anterior. | Molar. Posterior. |
|---|---|---|---|---|---|---|---|
| Six had . . . | 4 | 4 | 4 | 4 | 4 | 4 | 4 |
| One . . . . | 4 | 4 | | 4 | 2 | 4 | 3 |
| One . . . . | 4 | 4 | 1 | 4 | 1 | 4 | 2 |
| Two . . . . | 4 | 4 | 3 | 4 | 3 | 4 | |

In the Female Orphan Asylum, I examined nineteen girls at nine years old ; of these—

| GIRLS OF NINE YEARS OF AGE. | Incisor. | | Cuspid. | Bicuspid. | | Molar. | |
|---|---|---|---|---|---|---|---|
| | Central. | Lateral. | | Anterior. | Posterior. | Anterior. | Posterior. |
| Thirteen* had . | 4 | 4 | | | | 4 | |
| Five . . . | 4 | 2 | | | | 4 | |
| One . . . | 4 | | | | | 4 | |

Twenty-seven of thirteen years of age exhibited the following developments :—

| GIRLS OF THIRTEEN YEARS OF AGE. | Incisor. | | Cuspid. | Bicuspid. | | Molar. | |
|---|---|---|---|---|---|---|---|
| | Central. | Lateral. | | Anterior. | Posterior. | Anterior. | Posterior. |
| Fifteen had . . | 4 | 4 | 4 | 4 | 4 | 4 | 4 |
| Eleven . . | 4 | 4 | 3 | 4 | 4 | 4 | 3 |
| One . . . | 4 | 4 | 3 | 4 | 2 | 4 | 2 |

In the eastern St. Marylebone National School, there were seventy-three boys of nine years old ; of these—

| BOYS OF NINE YEARS OF AGE. | Incisor. | | Cuspid. | Bicuspid. | | Molar. | |
|---|---|---|---|---|---|---|---|
| | Central. | Lateral. | | Anterior. | Posterior. | Anterior. | Posterior. |
| Thirty-six had . | 4 | 4 | | | | 4 | |
| Six . . . | 4 | 3 | | | | 4 | |
| Seventeen . . | 4 | 2 | | | | 4 | |
| Three . . | 4 | 1 | | | | 4 | |
| Nine . . . | 4 | | | | | 4 | |
| Two . . . | 3 | 1 | | | | 4 | |

There were only two boys of thirteen years old in this school, they will therefore be included in the next table of that age.

Among the inmates of Christ's Hospital, I found twenty-four boys of nine years of age ; of these—

| BOYS OF NINE YEARS OF AGE. | Incisor. | | Cuspid. | Bicuspid. | | Molar. | |
|---|---|---|---|---|---|---|---|
| | Central. | Lateral. | | Anterior. | Posterior. | Anterior. | Posterior. |
| Sixteen had . . | 4 | 4 | | | | 4 | |
| Three . . | 4 | 3 | | | | 4 | |
| Three . . . | 4 | 2 | | | | 4 | |
| One . . . | 4 | 1 | | | | 4 | |
| One . . . | 3 | 3 | | | | 4 | |

And one hundred and twelve of the age of thirteen years, who presented the following appearances :—

* In two of these the lower cuspid teeth were making their appearance.

| Boys of thirteen years of age. | Incisor. Central. | Incisor. Lateral. | Cuspid. | Bicuspid. Anterior. | Bicuspid. Posterior. | Molar. Anterior. | Molar. Posterior. |
|---|---|---|---|---|---|---|---|
| Forty-six had . | 4 | 4 | 4 | 4 | 4 | 4 | 4 |
| Thirty-five . | 4 | 4 | 3 | 4 | 4 | 4 | 3 |
| Thirteen . . | 4 | 4 | 3 | 4 | 3 | 4 | 2 |
| Fifteen . . | 4 | 4 | 3 | 4 | 3 | 4 | 1 |
| Three . . . | 4 | 4 | 2 | 4 | 2 | 4 | |

With the examination of the boys in this institution, the inquiry is, for the present suspended, the results of the inspection of 1046 children being deemed sufficient to justify those views and opinions which are now submitted to the attention of the legislature. The statements exhibited in the foregoing tables, which have been prepared with the utmost accuracy and fairness, (a record of the name, exact age, and number and classes of teeth of each child, having been made and preserved,) are sufficiently convincing proofs of the superiority of this over every other physiological indication of age. By a reference to these, it will be seen, that of 457 boys of nine years of age—

| Boys of nine years of age. | Incisor. Central. | Incisor. Lateral. | Cuspid. | Bicuspid. Anterior. | Bicuspid. Posterior. | Molar. Anterior. | Molar. Posterior. |
|---|---|---|---|---|---|---|---|
| 220 had . . | 4 | 4 | | | | 4 | |
| Seventy-seven . | 4 | 3 | | | | 4 | |
| Ninety-one . . | 4 | 2 | | | | 4 | |
| Five . . . | 4 | 1 | | | | 4 | |
| Thirty-four . . | 4 | | | | | 4 | |
| Twenty . . | 3 | 3 | | | | 4 | |
| Ten . . . | 3 | | | | | 4 | |

Of 251 girls of nine years of age—

| Girls of nine years of age. | Incisor. Central. | Incisor. Lateral. | Cuspid. | Bicuspid. Anterior. | Bicuspid. Posterior. | Molar. Anterior. | Molar. Posterior. |
|---|---|---|---|---|---|---|---|
| 169 had . . | 4 | 4 | | | | 4 | |
| Forty-one . . | 4 | 3 | | | | 4 | |
| Twenty-seven . | 4 | 2 | | | | 4 | |
| Three . . | 4 | 1 | | | | 4 | |
| Four . . . | 4 | | | | | 4 | |
| Three . . | 3 | 3 | | | | 4 | |
| Four . . . | 3 | | | | | 4 | |

Of 227 boys of thirteen years of age—

| BOYS OF THIRTEEN YEARS OF AGE. | Incisor. Central. | Incisor. Lateral. | Cuspid. | Bicuspid. Anterior. | Bicuspid. Posterior. | Molar. Anterior. | Molar. Posterior. |
|---|---|---|---|---|---|---|---|
| 104 had . . | 4 | 4 | 4 | 4 | 4 | 4 | 4 |
| Fifty-seven . | 4 | 4 | 3 | 4 | 4 | 4 | 3 |
| Twenty-nine . | 4 | 4 | 3 | 4 | 3 | 4 | 2 |
| Thirty-three . | 4 | 4 | 3 | 4 | 2 | 4 | 1 |
| Four . . . | 4 | 4 | 2 | 4 | 1 | 4 | |

Of 111 girls of thirteen years of age—

| GIRLS OF THIRTEEN YEARS OF AGE. | Incisor. Central. | Incisor. Lateral. | Cuspid. | Bicuspid. Anterior. | Bicuspid. Posterior. | Molar. Anterior. | Molar. Posterior. |
|---|---|---|---|---|---|---|---|
| Seventy-one had . | 4 | 4 | 4 | 4 | 4 | 4 | 4 |
| Twenty-five . | 4 | 4 | 3 | 4 | 4 | 4 | 3 |
| Eight . . . | 4 | 4 | 3 | 4 | 3 | 4 | 2 |
| Three . . | 4 | 4 | 3 | 4 | 3 | 4 | 1 |
| Four . . . | 4 | 4 | 2 | 4 | 1 | 4 | |

Thus, then, it appears, that of 708 children of nine years of age, 389 would have been pronounced, on an application of this to be near the completion of their ninth year—that is, they presented the full developments of that age. But on the principle already stated, that of reckoning the fourth tooth as present where the three are fully developed, a still larger majority will be obtained, and, instead of 389, the proportion will be as follows:—Of 708 children, no less a number than 530 will be fully nine years of age. What, then, are the deviations exhibited by the remaining 178? They are the following:—126 would be pronounced eight years and six months, and the remaining 52 eight years of age; so that the extreme deviations are only twelve months, and these only in the inconsiderable proportion, (when compared with the results obtained by other criteria,) of 52 in 708.

Again, of 338 children of thirteen years of age, no less than 294 might have been pronounced, with confidence, to be of that age. The remaining forty-four would have been considered as follows:—thirty-six in their thirteenth, and eight near the completion of their twelfth year.

Upon these statements further comment would be unnecessary and impertinent; they are therefore submitted, without further observation, to the candid consideration, and favorable notice, of those who are anxious that nothing shall be left uninvestigated or unproved, that shall tend, in the slightest degree, to ameliorate the condition of that large, unprotected, and suffering class of the community, the factory children.

### ARTICLE IV.

### *Letter from* Dr. W. H. Dwinelle.

